# Effect of salting-out on distribution behavior of di(2-ethylhexyl) phthalate and its analogues between water and sediment

**DOI:** 10.1186/2193-1801-2-422

**Published:** 2013-08-30

**Authors:** Erini Yuwatini, Noriko Hata, Hideki Kuramitz, Shigeru Taguchi

**Affiliations:** Department of Environmental Biology and Chemistry, Graduate School of Science and Engineering for Research, University of Toyama, Gofuku 3190, Toyama, 930-8555 Japan

**Keywords:** Di(2-ethylhexyl) phthalate, Distribution, Sediment, Estuary, Hydrophobicity

## Abstract

A higher enrichment of organic pollutant, di(2-ethylhexyl) phthalate (DEHP) was found in estuary of *Oyabe* River and *Jinzu* River, Japan. Based on this, the distribution of DEHP between water and bed sediment was investigated as a model of organic pollutant through both the field investigation and laboratory experiment. The laboratory experiment was performed to examine the effect of seawater, organic matter in sediment and hydrophobicity (log *K*_*ow*_) of organic pollutants. The result showed that salting-out effect due to the high salinity in seawater and organic matter in sediment contributed towards the increasing of DEHP distribution between water and sediment. Furthermore, the hydrophobicity of organic pollutant also enhances the distribution between water and sediment to a higher magnitude in the presence of seawater.

## Introduction

Phthalate esters are widespread environmental contaminants (Giam et al. 1978; Thurén and Larsson 1990; Ma et al. 2013). They have a wide variety of industrial, agricultural and domestic applications but the most important use is as non-reactive plasticisers that improve the flexibility and workability of plastic materials, especially for polyvinyl chloride. Phthalates only exhibit subtle toxicity to aquatic organisms (Giam et al. 1984; DeFoe et al. 1990). Their bioaccumulation through the aquatic food chain is limited by biotransformation of phthalates, which increases with trophic level (Staples et al. 1997). However, phthalates are one of the suspected endocrine disrupting chemicals that require our concern (Jobling et al. 1995; Barton and Andersen 1998; Okamoto et al. 2011). Some investigations to study the effect of phthalates on human (Latini et al. 2004) and animal (Maire et al. 2005) have been performed.

One of the most important phthalates is di(2-ethylhexyl) phthalate (DEHP). There are many studies that have reported on the concentration of DEHP in aquatic environment. Fresh water resources such as river and lake were polluted with DEHP released from industries and urban area (Vitali et al. 1997). Pollution of DEHP was also caused by sewage treatment plant (Marttinen et al. 2003) and landfill leachate (Asakura et al. 2004). Our previous field investigation showed that DEHP from domestic waste water significantly polluted bed sediment in river (Yuwatini et al. 2006). The laboratory examination of DEHP confirmed its adsorptive potential from water to sediment and also slow degradation in river sediment. The slow degradation under both aerobic and anaerobic conditions attributed to the forming particle-bound complex (Johnson et al. 1984; Roslev et al. 1998) that may cause the bed sediment to act as long-term sink and secondary source of DEHP.

One of the areas in aquatic environmental pollution that has been receiving much attention is estuary. Previous reports have indicated that the concentrations of DEHP in bed sediment at estuary are greater in magnitude than that in the water column (Ray et al. 1983; Preston and Al-Omran 1989). Findings also have shown the interaction of DEHP with sediment particle in simulated estuary system as reported by Zhou and Liu (2000). Turner and Rawling (2000) have investigated the particles-water interactions of DEHP in river water and sea water by monitoring uptake of a radiolabelled analogue by natural estuarine particles. Their laboratory experiments have shown that more than 50% of DEHP discharged to a catchment maybe retained in the estuary, at least with respect to a timescale equivalent to the estuarine particle residence time.

The studies on the behavior of organic pollutants distribution in estuary region were mostly performed under simulated experiment in laboratory. However, in order to better understand the behavioral distribution, it is essential for the field and laboratory investigation to be carried out. The comparison of methods is critical in assessing the influence of the organic characteristics such as hydrophobicity at the estuary distribution.

In this study, the distribution of DEHP between water and sediment and also suspended solid at estuary were investigated to further enhance our understanding on the transport and fate of DEHP in aquatic environment. The experiments were performed to clarify (i) the effect of seawater, (ii) the organic matter in sediment, and (iii) the hydrophobicity of organic pollutant on the distribution between water and sediment. Nitrobenzene, α-naphtoquinoline, di-n-butyl phthalate (DnBP), di-n-hexyl phthalate (DnHP), di-n-octyl phthalate (DnOP) di-n-pentyl phthalate (DnPP) and DEHP were used as model chemicals which have a different octanol/water partition constant (*K*_*ow*_).

## Material and methods

### Reagents and apparatus

Phthalate esters (DEHP, DnBP, DnHP, DnOP and DnPP) standard solution was obtained from Tokyo Chemical Industry, Japan. α-Naphthoquinoline standard solution and nitrobenzene standard solution were purchased from Wako chemicals, Japan. The standard solution of phthalate esters and α-naphthoquinoline were diluted with methanol. The nitrobenzene standard solution was diluted in distilled water. 4-Trifluoromethylaniline (ABTF) and dodecylbenzene sulfonate sodium salt (DBS) used to form ion-associate organic phase for DEHP determination in water sample were obtained from Tokyo Chemical Industry, Japan. A 0.1 M ABTF solution was prepared by diluting in 2 M hydrochloric acid. Five mM DBS solution was prepared with distilled water. 2-Methoxy ethanol, acetone, acetonitrile, methanol and n-hexane (Wako Chemicals, Japan) were used analytical grade.

A glass fiber filter (GC50, 0.45 μm pore size, 47 mm in diameter) purchased from Advantec Toyo, Japan was used to filter the water samples and to prepare sediment samples. Oasis HLB cartridge (Waters, USA) was used to clean up the extract from sediment sample. A centrifuge (SCT 5BB, Hitachi, Japan) was used for water and sediment sample preparation. The determination of phthalate esters was performed by HPLC with octadecyl silica column (ODS-80TS, 4.6 mm × 250 mm, Tosoh TSK Gel, Japan), column heater (U-620, Sugai, Japan) and an intelligent HPLC pump (PU-980, Jasco, Japan) connected to a multi wavelength detector (MD-1515, JASCO, Japan). An ion chromatography was performed by 761 Compact IC (Metrohm, Switzerland) to determine ion chloride in water sample. A microscope (IX/70, Olympus, Japan) equipped with zoom Olympus camera was used to determine particle size of sediment. Elemental analyzer (CN Corder MT-700, Yanagimoto, Kyoto, Japan) was used to determine the carbon content (%C) in sediment.

### Study sites and sampling

**Figure 1:**
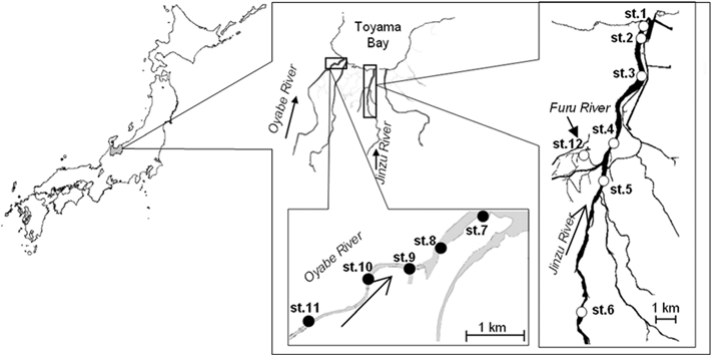
**Location of investigation.** Station 1 to 6 are in *Jinzu* River and station 7 to 11 are in *Oyabe* River. Station 12 is in *Furu* River which is running across an urban area and flowing to *Jinzu* River.

The map of the investigation locations are shown in Figure 1. The sampling areas included *Jinzu* River and *Oyabe* River in *Toyama* prefecture which consists of a length and average drainage area of 135 km; 2720 km^2^ and 68 km; 667 km^2^ respectively. Stations 1 to 6 are located on *Jinzu* River with a distance of 20 km from estuary and a flow amount from 44 to 1,630 m^3^/s. The remaining stations (Station 7 to 11) are situated at *Oyabe* River which is 6.1 km from estuary, from 50 to 210 m in width and flow amount of 16 m^3^/s to 600 m^3^/s. Water and sediment samples were collected every two months from January 2002 to December 2003 in *Jinzu* River and from November 2003 to August 2004 in *Oyabe* River. Sediment sample was also collected from station 12 that is located at the domestic waste water discharge point of *Furu* River. *Furu* River is a small river running through the *Toyama* City.

### Sample preparation

The river water samples were collected in dark bottle and kept at a cool box during transportation to the laboratory. In laboratory, water samples were filtered through glass fiber filter and kept at 4°C in dark condition before analysis. Sediment samples were collected in stainless steel container. The samples were centrifuged to squeeze water from the sediment and dried at 110°C for 2 hours. The dried sediment sample was sieved by 16 mesh. Suspended solids were isolated from 3.5 to 7.0 L of river water by centrifugation at 3000 rpm for 15 min. The obtained suspended solids (about 0.2 g) were dried at 110°C for 2 hours. All of the equipments used in the experimental were rinsed with acetone before use to avoid contamination.

### Analytical procedures

The analytical procedure using the formation of ion-associate organic phase for the determination of DEHP in water samples is similar to our previous report (Hata et al. 2004). Highly pre-concentrated DEHP from sample was injected into HPLC system with acetonitrile : 0.02 M KCl solution = 90:10 as a mobile phase. The detection limit for this method is 0.07 μg/L calculated three times of the standard deviation of blank solution.

The determination of DEHP in sediment samples were carried out as follow. About 25 g of dried sediment was treated in 40 mL acetone under ultrasonic radiation (120 W of output; SU-30, Shibata, Japan) for 4 times with each measuring to a time period of 15 min. The crude extracts in acetone were filtered through glass fiber filter and acetone was removed by a rotary-evaporator. n-Hexane was added to dissolve the residue and washed with water. The n-hexane phase was evaporated again, and then the residue was dissolved with methanol. After the solution was diluted with water (methanol solution : water = 1:40), the obtained solution was passed through the solid phase extraction device of HLB cartridge. The DEHP retained on the cartridge was eluted with methanol, and 20 μL of the elute was injected into HPLC column with a mixture of acetonitrile and water (acetonitrile : water = 90:10) as a mobile phase. The limit of detection in this method was 20 μg/kg calculated from five time of noise in blank solution.

All dried suspended solids obtained from river water were treated with 5 mL acetone under ultrasonic radiation for 4 times with each measuring to a time period of 15 min. The crude extracts were collected and DEHP analysis was performed similar to the sediment procedure.

All other phathalate esters (DnBP, DnHp, DnOP and DnPP) in water and sediment samples were determined by the similar procedure of DEHP in water and sediment.

Nitrobenzene and α-naphthoquinoline contained in water samples was directly injected into the HPLC (ODS column, acetonitrile : water = 70:30) with the same condition as the determination of DEHP. Nitrobenzene and α-naphthoquinoline in sediment samples (0.5 g) was determined by extracting the sediment with acetone under ultrasonic radiation for 4 times with each measuring to a time period of 5 min. The extracts were evaporated followed by dilution in methanol before injection into HPLC.

In order to determine the carbon content in sediment, elemental analysis was performed on the dried sediment samples (1 g).

### Investigation of DEHP distribution between water and sediment

The effect of seawater on the behavioral distribution of DEHP onto sediment were investigated by placing 0.5 g of each dried sediment into several centrifuge tubes containing 50 mL of DEHP aqueous solution spiked with 0, 10, 20, 40 and 50% of seawater that was collected from *Toyama* Bay. In order to avoid the biodegradation during experiment, both of the seawater and distilled water were irradiated with UV 185/254 nm lamp (12 W) for 30 min before experiment (Iwaguchi et al. 2000,2002). All the tubes were shaken with 70 strokes per min at 25°C for more than 20 hours to reach the equilibrium state in which the separated water and sediment were then examined for DEHP concentrations respectively. Sediments collected from st.2, st.6, st.7, st.11 and st.12 were separately examined with the same procedure.

## Results and discussion

### DEHP concentration in *Jinzu* River and *Oyabe* River

**Figure 2:**
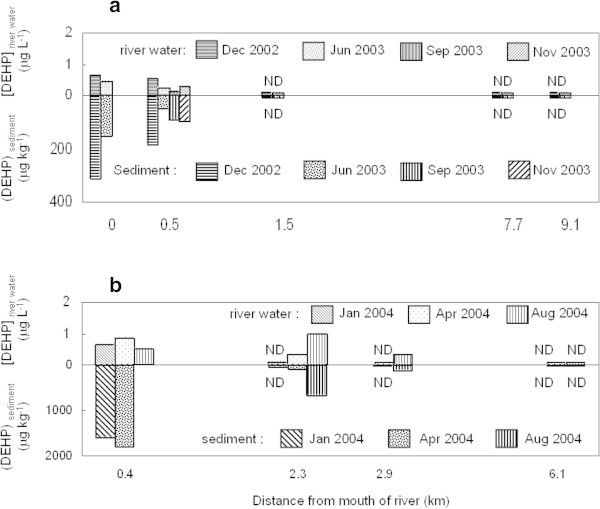
**DEHP concentration in river water and sediment of (a)*Jinzu*River and (b)*Oyabe*River.**

The DEHP concentrations in water and sediment samples from *Jinzu* River and *Oyabe* River are shown in Figure 2. The x-axis shows the distance from river mouth to upper stream direction. The y-axis shows the concentration of DEHP in water (upper part of figure) and sediment (lower part of figure). The found concentration of DEHP ranges from less than 0.07 to 0.6 μg/L and less than 20 μg/kg to 300 μg/kg in *Jinzu* River, and less than 0.07 to 1 μg/L and less than 20 μg/kg to 1800 μg/kg in *Oyabe* River. In the both of rivers, DEHP concentrations in sediment from locations points that was closer to estuary (up to 3 km from the sea) were significantly higher than others. The ratio of DEHP concentrations in sediment to river water was higher in magnitude by the distance leading to estuary. It is considered that the effect of seawater and sediment characteristics contribute towards the enhancement of the DEHP distribution between water and sediment and this will be elaborated in the next section.

### Distribution of DEHP between water and sediment

The distribution of organic pollutants between water and sediment can be expressed by the ratio of its concentration in sediment to water which is generally defined by the following equation:1

Where, *D*_*pollutant*_ is distribution efficiency of the organic pollutant X. (X)_s_ and [X]_w_ represent the concentration of organic pollutant adsorbed on sediment (in μg/kg sediment) and the remain in aqueous solution (in μg/L). Here, the distribution of organic pollutant between water and sediment, *D*_*pollutant*_ is represented as *D*_*pollutant,exp*_ for the experiment in equilibrium state, and (X)_s*,field*_/[X]_w,*field*_ for distribution between sediment and water in field investigation. In the case of DEHP as the object, *D*_*pollutant*_ is represented as *D*_*DEHP,exp*_, (DEHP)_s*,field*_/[DEHP]_w,*field*_ for sediment and river water whereas (DEHP)_ss*,field*_/[DEHP]_w,*field*_ for suspended solids and river water.

**Figure 3:**
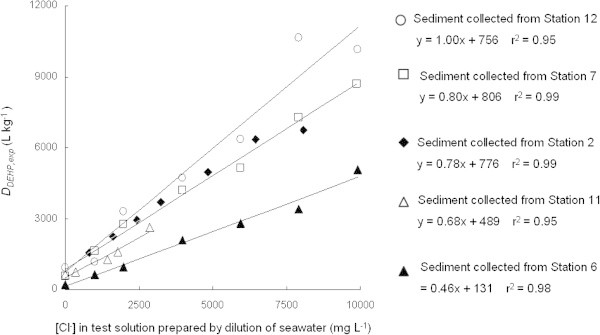
**Effect of seawater*vs*.*D***_***DEHP,exp***_**on equilibrium state for various sediments.**

As for the investigation on the effect of seawater on *D*_*DEHP,exp*_, the collected sediment samples from some of sampling sites were used in the laboratory experiment. The result showed that *D*_*DEHP,exp*_ increased with increasing the concentration of seawater that was determined by the concentration of Cl^-^ in the test solution (Figure 3). This is due to the salting-out effect of the salinity in seawater which caused a decrease of DEHP solubility in water, thus giving a higher tendency of its adsorption onto sediment particle. So the DEHP distribution between sediment and water defined as *D*_*DEHP,exp*_ was directly affected with seawater existence. The relationship between [Cl^-^] and *D*_*DEHP,exp*_ was linear with correlation, r^2^ more than 0.95. Therefore, *D*_*DEHP,exp*_ from equation 1 would be extended by [Cl^-^] concentration factor as follows:2

Where, *a* is the constant related to seawater effect measured as Cl^-^ concentration and *b* is the constant relevant to *D*_*DEHP,exp*_. In this study, no significant difference was found between estuary sediments from st.2 and st.7. It is probably that both sediments collected from the areas around the estuary have similar characteristics. The sediments from both st.2 and st.7 appeared in dark color sand and consisted partially of silt, with particle size less than 0.1 mm. As for the relationship between [Cl^-^] and *D*_*DEHP,exp*_, parameters of sediment samples taken from st.6, st.11 and st.12, it exhibited a difference in the slope. The sediment taken at st.6 and st.11 from *Jinzu* River and *Oyabe* River appeared in brown color with partial silt and particle size measured less than 1.0 mm. The sediment from st.12 that was located at domestic wastewater in *Furu* River discharge point appeared in dark color s, containing mostly of sand and partially of silt, with particle size of sediment less than 0.5 mm. It is assumed that smaller sediment particle size will give a wider surface area for the adsorption in every unit of particle weight. On the contrary, a smaller particle size of sediment would result in a higher *D*_*DEHP, exp*_. These findings were consistent with sediment from st.2, st.6, st.7 and st.11. Even though the sediment’s particle size from station 12 was bigger than st.2 and st.7, it still exhibited a higher *D*_*DEHP, exp*_. This is probably due to the characteristic of the sediment itself, where by the influence of the organic matter that adsorbed on the sediment surface affects the distribution of DEHP between sediment and water much more than particle size of sediment.

**Figure 4:**
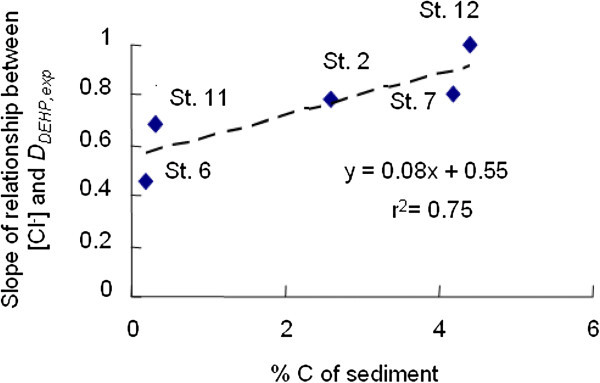
**Plots of carbon content (%) in sediment*vs*. slope resulted from relationship between [Cl**^**-**^**] and*D***_***DEHP, exp***_**from Figure**3**.**

Carbon content in sediment was considered to be relevant with organic matter adsorbed on the sediment surface. In assumption that organic matter adsorbed on surface of sediment behaves like organic sorbent and the hydrophobic neutral organic pollutants in water is adsorbed into organic matrix. The percentages of carbon content found in sediments were 2.6, 0.2, 4.2, 0.3 and 4.4% for st.2, st.6, st.7, st.11 and st.12, respectively. The relationship between carbon content (%) in sediment and slope obtained from the relationship between [Cl^-^] and *D*_*DEHP,exp*_ is shown in Figure 4. The correlation obtained (r^2^ = 0.75) showed that content of carbon in sediment also contributed to an increase of seawater effect on *D*_*DEHP, exp*._ The intercept on coordinate showed that DEHP adsorption on sediment occurred when %C = 0, as surface phenomenon.

**Figure 5:**
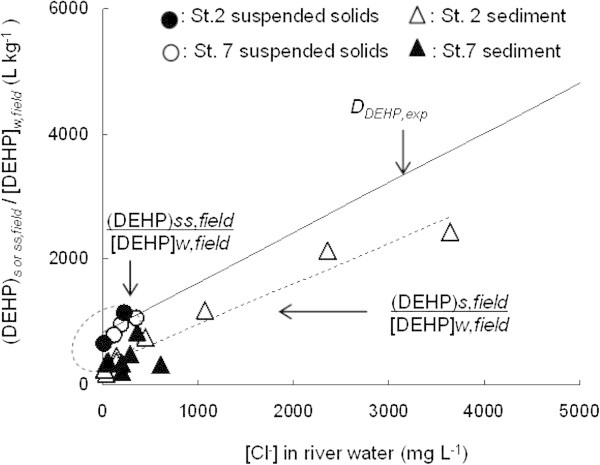
**Comparison of [Cl**^**-**^**] in river water versus (DEHP)**_**s or*ss*,*field***_**/[DEHP]**_***w,field***_**at*Jinzu*River and*Oyabe*River.** Upper lines is the result of *D*_*DEHP,exp*_ from station 2 and 7 from Figure 3. The inside of circle broken line is (DEHP)_*ss*,*field*_/[DEHP]_*w,field*_. Lower broken line is (DEHP)_s,*field*_/[DEHP]_*w,field*_.

The effect of seawater on (DEHP)_s,*field*_/[DEHP]_*w,field*_ and (DEHP)_ss,*field*_/[DEHP]_*w,field*_ was also investigated. Figure 5 shows the plots of (DEHP)_s,*field*_/[DEHP]_*w,field*_ and (DEHP)_ss,*field*_/[DEHP]_*w,field*_*vs*. [Cl^-^] in *Jinzu* River and *Oyabe* River water. The result indicates that both (DEHP)_s,*field*_/[DEHP]_*w,field*_ and (DEHP)_ss,*field*_/[DEHP]_*w,field*_ increased with increasing [Cl^-^] in river water. (DEHP)_ss,*field*_/[DEHP]_*w,field*_ appeared to be closer to the *D*_*DEHP,exp*_. Thus, suggesting that the suspended solids that flow with river water (from the river to the sea and vice versa) appears to provide a result that is closer to *D*_*DEHP,exp*_.

### Relationship between log *K*_*ow*_ for organic pollutants and distribution between water and sediment (*D*_*pollutant,exp*_)

The effect of seawater on the behavior of several organic pollutants was investigated by the experimental simulation in laboratory. It has been reported that the increment of nitrobenzene adsorption in marine sediment was about 30% in medium of seawater than in distilled water (Zhao et al. 2003). The field investigation on the adsorption of fluoranthene and pyrene on sediment were not affected in presence of seawater (Zhao et al. 1998), even though the batch experiment adsorption of fluoranthene and phenanthrene were increased up to 34% in seawater (Tremblay et al. 2005). The adsorption of dibutyltin in sediment was decreased in presence of seawater medium (Hoch et al. 2003). Therefore, it can be expected that seawater effect on *D*_*pollutant,exp*_ could be caused by hydrophobicity of organic pollutant.

Based on these facts, the *K*_*ow*_ for organic pollutant was considered as the factor affecting its distribution between sediment and water. Seven organic pollutants were chosen for the examination based on the different values of *K*_*ow*_. The tested compounds were nitrobenzene (log *K*_*ow*_ = 1.85), α-naphthoquinoline (log *K*_*ow*_ = 3.43) and several phthalate esters, such as DnBP (log *K*_*ow*_ = 4.50), DnPP (log *K*_*ow*_ = 5.62), DnHP (log *K*_*ow*_ = 6.82), DEHP (log *K*_*ow*_ = 7.27) and DnOP (log *K*_*ow*_ = 8.10) (Hansch et al. 1995; Ellington and Floyd 1996).

**Figure 6:**
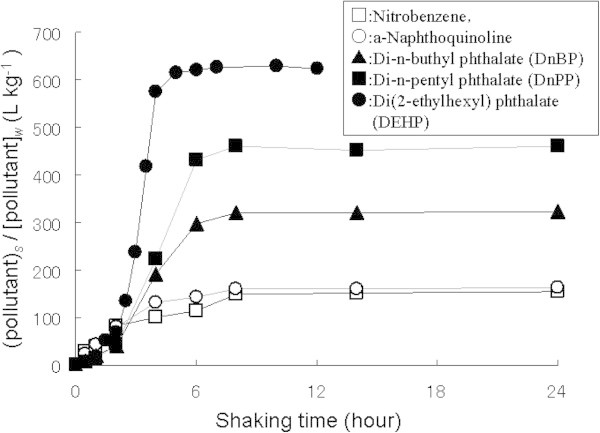
**Kinetics of adsorption for organic pollutants.**

The kinetics of adsorption experiment was prior investigated for each compound. As shown in the results in Figure 6, the compounds with higher hydrophobicity give shorter shaking time to reach the equilibrium state than the lower ones such as in the case of DEHP and nitrobenzene; it takes about 4 hours and 8 hours respectively to reach the equilibrium state. It is probably that the compound with higher hydrophobicity was rapidly adsorbed and then retained on sediment surface. On contrary, the compound with lower hydrophobicity probably was not easy to retain in sediment surface due to its solubility in water phase.

**Figure 7:**
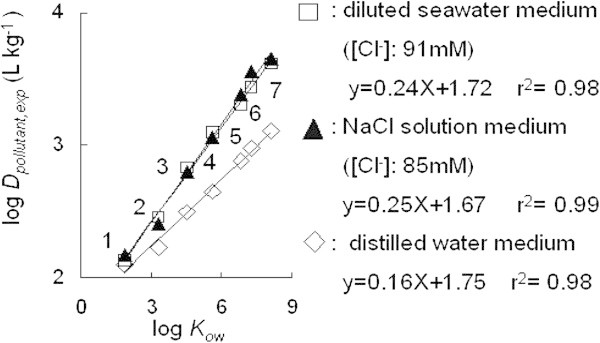
**Plots of Log*K***_***ow***_**of organic pollutant versus logarithmic*D***_***pollutant,exp***_**. (1)** Nitrobenzene, **(2)** α-naphthoquinoline, **(3)** DnBP, **(4)** DnPP, **(5)** DnHxP, **(6)** DEHP, **(7)** DnOP.

The *D*_*pollutant,exp*_ of organic pollutant with log *K*_*ow*_ between 2 to 8 were then examined. To confirm the salting-out effect on *D*_*pollutant,exp*_, the experiment was undertaken in NaCl solution media, diluted seawater, and also distilled water. Figure 7 shows that *D*_*pollutant,exp*_ for tested compounds were enhanced with increasing the hydrophobicity (log *K*_*ow*_). The increment of *D*_*pollutant,exp*_ in media of NaCl solution and diluted seawater were higher than in the medium of distilled water. The obtained relationship between log *D*_*pollutant,exp*_ and log *K*_*ow*_ was a linear correlation. Therefore, *D*_*pollutant,exp*_ from equation (1) would also be extended by log *K*_*ow*_ factor as follows:3

Where, *D*_*pollutant,exp*_ is the distribution between water and sediment in equilibrium state, *c* and *d* are a constants related to *D*_*pollutant,exp*_ which depends on the hydrophobicity of compound.

The slope increased up to 1.5 times in both media of seawater and NaCl solution than in the case of distilled water. This suggests that aqueous solubility of the compound with higher hydrophobicity decreased due to salting-out effect in both media of NaCl solution and diluted seawater. Both media (diluted seawater and in NaCl solution) enhanced the adsorption potential of hydrophobic organic pollutant from water to sediment.

## Conclusions

In this study, we concluded that the distribution of DEHP from water to sediment in estuary was increased due to salting-out effect which was confirmed through the experiment in both media of diluted seawater and NaCl solution. Organic matter in sediment contributed to the increment of distribution of DEHP from water to sediment. With increasing the hydrophobicity of organic pollutants, the distribution between water and sediment was in higher magnitude with the presence of seawater.
